# Enhanced Prediction of Cardiovascular Disease Through Integrated Machine Learning Models Combining Clinical and Demographic Characteristics

**DOI:** 10.3390/diagnostics16101572

**Published:** 2026-05-21

**Authors:** Zhe Zhang, Dengao Li, Jumin Zhao, Huiting Ma, Fei Wang, Qinglian Hao

**Affiliations:** 1 College of Integrated Circuits, Taiyuan University of Technology, Taiyuan 030024, China; 2 College of Computer Science and Technology (College of Data Science), Taiyuan University of Technology, Taiyuan 030024, China; 3 Cardiovascular Medicine, Shanxi Cardiovascular Hospital, Taiyuan 030024, China; 4 Cardiovascular Medicine, Shanxi Provincial Integrated TCM and WM Hospital, Taiyuan 030024, China

**Keywords:** heart failure, machine learning, predictive analytics, clinical indicators, demographic characteristics

## Abstract

**Background/Objectives:** Heart failure (HF) remains a major cause of global mortality and morbidity; it is, therefore, of paramount importance that diagnosis and prognostication are made timely in order to better improve outcomes and reduce healthcare expenditure. This research presents a novel predictive model of heart failure that combines clinical criteria with demographic factors in order to maximize predictive performance and act as a reliable tool for individualized healthcare intervention. **Methods:** Complex machine learning techniques, including decision trees, random forest, and deep learning, are applied in analyzing a large dataset of subjects with heart failure. We collected a diverse dataset comprising clinical indicators such as echocardiographic data, biomarkers, electrocardiogram (ECG) features, and demographic information. Data preprocessing techniques, such as feature normalization and handling of missing values, were applied to ensure the integrity and reliability of the dataset. **Results:** The results indicate that integrating both clinical indicators and demographic characteristics significantly improves the predictive power of the model, compared to models based on clinical indicators alone. Specifically, the hybrid model demonstrated a superior ability to predict short- and long-term outcomes in heart failure patients, offering enhanced accuracy in risk stratification and prognosis prediction. **Conclusions:** This research highlights the potential of artificial intelligence (AI) and machine learning in revolutionizing heart failure care by providing healthcare professionals with more accurate, data-driven decision support tools. The proposed model not only holds promise for clinical applications but also offers insights for future research into personalized medicine.

## 1. Introduction

Cardiovascular diseases, particularly coronary artery disease, remain a leading cause of global morbidity and mortality. Early detection of heart disease using clinical and demographic indicators is essential to improve patient outcomes and optimize healthcare resources [1,2]. Present statistical projections indicate that the number of patients with heart failure in the United States is also projected to grow from approximately 6.7 million in 2024 up to 8.7 million in 2030, and up to 11.4 million in 2050 [3]. HF is the leading reason for hospitalization in those over the age of 65, and healthcare spending on heart failure accounts for almost 2% of overall healthcare expenses in developed countries [4]. Unfortunately, the case-fatality rate during hospital discharge of heart failure is reported to be 10.4% in the first 30 days, 22% in one year, and is expected to increase up to 42.3% in five years of follow-up [5]. While the incidence of heart failure has stabilized or actually decreased in developed nations, its prevalence is still on the rise due mostly to an aging population, improved quality of management of cardiac diseases, and increased survival rates of patients with heart failure [6,7].

Prompt diagnosis and accurate prediction of heart failure are necessary for improved patient outcomes and reduced healthcare spending [8]. Despite significant developments in understanding the underlying fundamental mechanisms of heart failure, the diagnostic and treatment processes remain challenging due to their complexity and multifactorial nature [9]. In the past, the evaluation and treatment of heart failure have relied in large part on clinical measurements of ejection fraction, serum biomarkers, and electrocardiographic interpretation. However, these methods have inherent shortfalls in terms of predictive performance and usually are not able to account for the complexity of the onset and course of heart failure [10]. Over the last decade, developments in machine learning and artificial intelligence (AI) technology have created unprecedented opportunities for an evolutionary redefinition of cardiovascular medicine [11]. Studies have shown that machine learning-based algorithms have remarkable strength with high-dimensional and complex data, being able to identify relationships that are not visible with conventional statistical methods [12]. Numerous investigations have shown that machine learning has a lot of promise in forecasting the incidence, course, and outcome of heart failure on the basis of clinical information [13].

In spite of the significant progress that earlier research has made in applying machine learning methods for the prediction of heart failure, most of the earlier investigations have focused primarily on clinical variables, possibly ignoring the significant impact that demographic variables could have [14]. Demographic variables are age, sex, race, and socioeconomic status, and they play an essential role in evaluating the probability of heart failure onset, the severity of the condition, and the success of the treatment opportunities. The integration of clinical data together with demographic details is expected to further improve the accuracy of heart failure models, and in turn, enhance individualized risk assessment for patients [15]. The principle of personalized medicine, or the individualization of healthcare practices for the unique needs of individual patients, has received considerable attention in recent research in academia. In the case of heart failure, personalized medicine has the potential to move beyond the traditional “one-size-fits-all” practice by providing optimized, individualized treatment plans that correspond with the unique characteristics of individual patients. By including demographic parameters in clinical prediction models, machine learning algorithms have the potential to refine the detection of heterogeneous patient populations with dissimilar risks and responses, leading to the creation of more targeted patient management regimens.

This study is designed to fill the current gap by creating a novel predictive model of heart failure that combines clinical predictors with demographic information. The research hopes to create a model with high predictive power and clinical utility, serving as a valuable tool in individualized medicine through the use of recent machine learning techniques, namely decision trees, random forests, and deep learning platforms. The combination of demographic information with conventional clinical indicators not only increases the promise of predictive accuracy but also enhances an understanding of the complex interaction between patient attributes and outcomes of heart failure. The implications of this research are far-reaching for clinical practice, health policy, and ongoing research in heart failure treatment, with the potential for supporting more accurate and personalized risk stratification that could help clinicians identify high-risk individuals earlier, refine treatment adjustments, and better direct healthcare resources.

## 2. Materials and Methods

### 2.1. Dataset

This study utilized the Heart Failure Prediction Dataset published by fedesoriano on Kaggle in September 2021. The dataset contains 920 patient records with demographic and clinical information, providing a robust foundation for developing predictive models that integrate both clinical indicators and demographic characteristics. To mitigate overfitting, dropout layers (rate = 0.3) and early stopping were applied during training. Model complexity was controlled through validation performance monitoring.

The dataset comprises 725 males and 195 females of various ages, with 458 males and 50 females diagnosed with heart disease, while 267 males and 145 females were classified as normal, as documented in the recent literature. Eleven clinical features were recorded for each patient: Age, Sex, ChestPainType (typical angina, atypical angina, non-anginal pain, or asymptomatic), RestingBP (resting blood pressure in mm Hg), Cholesterol (serum cholesterol in mg/dL), FastingBS (fasting blood sugar > 120 mg/dL), RestingECG (resting electrocardiogram results), MaxHR (maximum heart rate achieved), ExerciseAngina (exercise-induced angina), Oldpeak (ST depression induced by exercise relative to rest), and ST_Slope (slope of the peak exercise ST segment). The target variable, HeartDisease, is a binary indicator denoting the presence (1) or absence (0) of heart disease. Only variables present in the Kaggle Heart Disease dataset were included in the analysis. Any previously reported variables not in the dataset have been removed.

For this study, the dataset was randomly divided into training (70%), validation (15%), and testing (15%) sets, ensuring balanced distribution of the target variable across all subsets. This partitioning approach facilitates robust model development, hyperparameter tuning, and unbiased performance evaluation. Missing values were handled using median imputation due to its robustness to outliers and simplicity for small datasets. Multiple imputation methods were not applied in this study [10].

**Figure 1:**
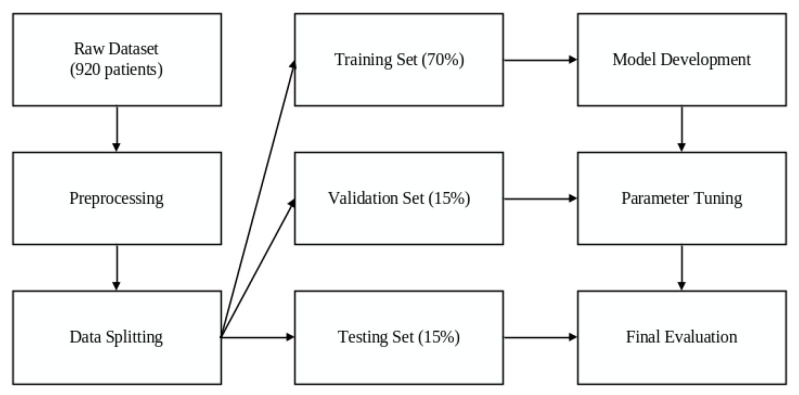
Data processing workflow.

The dataset for training allowed for the creation of predictive models, whereas the dataset for validation was utilized for hyperparameter tuning and preventing overfitting. At the same time, the test dataset was held back for final performance assessment, ensuring that the models reflected generalizability on new, unseen data (Figure 1).

### 2.2. Machine Learning Algorithms

The creation of a predictive model of heart failure required the blending of diverse clinical and demographic factors, thus requiring the use of sophisticated predictive analytics methods. This model was created through an ensemble technique that merged decision trees, random forests, and deep learning methods, each of which provides unique advantages in controlling complex medical systems in terms of multidimensional characteristics and interdependent relationships.

The deep neural network model was trained using the binary cross-entropy loss function defined as follows:

*L* = −[*y* log(*p*) + (1 − *y*) log(1 − *p*)].

where *y* represents the true label and *p* represents the predicted probability. This formulation ensures appropriate optimization for binary classification tasks.

The algorithm chosen as the foundation for this research was decision trees, due to their simplicity and effectiveness in handling both categorical and numeric data as well as requiring limited preprocessing. The Classification and Regression Tree algorithm was used, with Gini impurity being the splitting criterion, allowing for recursive partitioning of the feature space and ultimately creating a tree structure that realizes the decision rules. This resulted in clear decision paths that can be visualized, allowing for increased transparency in the prediction process for clinicians. Pruning methods were also applied in order to counteract overfitting, with the best parameters determined by cross-validation on the validation set.

Random forests were employed to enhance prediction accuracy by addressing the inherent limitation of decision trees’ sensitivity to small variations in the training data. This ensemble method constructed 100 decision trees with bootstrap sampling and random feature selection at each split, where each tree voted for the final classification outcome. The number of features considered at each split was set to the square root of the total number of features, as recommended in the literature. Random forests provided not only improved accuracy but also robust feature importance metrics that helped identify the most influential predictors of heart failure [16].

Deep learning algorithms were implemented using a neural network architecture specifically designed for tabular data containing both clinical indicators and demographic characteristics. The network consisted of four hidden layers with 128, 64, 32, and 16 neurons, respectively, utilizing ReLU activation functions to introduce non-linearity. Dropout layers with a rate of 0.3 were inserted between hidden layers to prevent overfitting. The output layer used a sigmoid activation function to produce probability values for binary classification. The model was trained using the Adam optimizer with a learning rate of 0.001 and binary cross-entropy as the loss function. Early stopping was implemented with a patience of 20 epochs to prevent overfitting, using the validation loss as the monitoring metric [17].

To systematically compare algorithm performance, a comprehensive evaluation framework was established. The models were assessed using multiple performance metrics including accuracy, precision, recall, F1-score, and area under the receiver operating characteristic curve (AUC-ROC). In addition, confusion matrices were created to demonstrate true positives, true negatives, false positives, and false negatives for every model. The confusion matrices for the decision tree, random forest, and deep neural network models are provided in Table S1, Table S2, and Table S3, respectively. Statistical performance difference determination between the models was performed using McNemar’s test, with a significance of 0.05. Feature importance analysis was conducted in order to ascertain significant clinical predictors and demographic predictors of heart failure prediction, with methods suited for each algorithm: Gini importance for decision trees, permutation importance for random forests, and integrated gradients for the deep learning model.

### 2.3. Model Evaluation

The evaluation of the heart failure prediction models was conducted using a multifaceted assessment approach. Key indicators included precision, recall, F1-score, and the area under the receiver operating characteristic curve (AUC-ROC) in addition to overall accuracy. As calculated through the determination of a manual success evaluation, accuracy relied on the total number of correct prediction outcomes, whereas precision and recall addressed prediction accuracy and completeness in the positive class, respectively. F1-score served as an aggregate measure of model performance, alongside AUC-ROC, which illustrated the degree of class discrimination achievable with varying probability thresholds.

In order to capture the complete picture, a 10-fold cross-validation approach was utilized while developing the model, with the training dataset portioned into 10 folds. The models were trained on 9 folds and validated on the remaining fold which minimized overfitting while creating realistic performance estimates. The final evaluation of the model was performed using the withheld testing dataset to assess actual deployment performance without any tailoring to the model evaluation. To avoid data leakage, 10-fold cross-validation was applied only within the training dataset for hyperparameter tuning. Final model performance was evaluated using a held-out test set. Dropout and early stopping were used to reduce overfitting. Model performance metrics are reported as mean values across cross-validation folds. Variability was assessed to ensure robustness of results.

A comparison study was carried out to assess the performance of adding clinical indicators to demographic features. For each algorithm, three versions of the models were created: one with only clinical indicators, one with only demographic features, and one that combines both feature types. This comparison quantified the improvement achieved through feature integration and identified the most effective feature combination for heart failure prediction. Feature importance analysis was conducted using algorithm-specific methods to identify the most influential predictors and understand the relative contribution of different clinical and demographic characteristics.

## 3. Results

### 3.1. Performance Comparison of Machine Learning Algorithms

The evaluation of the three machine learning algorithms implemented in this study—decision trees, random forests, and deep neural networks—revealed significant differences in their predictive performance for heart failure. This pattern of relative performance was consistent across all evaluation metrics, demonstrating the superior capabilities of more complex ensemble and deep learning methods over simpler algorithms for this particular prediction task. The performance metrics of each algorithm are shown in Table 1, including accuracy, precision, recall, F1-score, and area under the ROC curve (AUC-ROC).

Analysis of the confusion matrix for the deep neural network model showed that, out of 138 test samples, 66 heart disease cases were correctly classified (true positives) and 62 non-heart disease cases were correctly identified (true negatives). The model produced five false positives and five false negatives. These results correspond to an overall accuracy of approximately 92.7%, consistent with the reported performance metrics. The random forest algorithm produced slightly more misclassifications with 16 false negatives and 14 false positives, while the decision tree model had the highest error rates with 27 false negatives and 23 false positives.

Statistical significance testing using McNemar’s test confirmed that the performance differences between all pairs of algorithms were statistically significant (*p* < 0.05), validating that the observed performance advantages of the deep neural network and random forest over the decision tree were not due to chance. The superior performance of the deep neural network can be attributed to its ability to model complex non-linear relationships between predictors and outcomes, as well as its capacity to automatically identify and learn from high-level interactions between clinical indicators and demographic characteristics. In the same manner, the estimated effectiveness of the random forest is probably due to the fact that it is an ensemble method which combines many decision trees to reduce overfitting and variance.

The analysis shows that while all three algorithms make quite good predictions for heart failure, cutting-edge methods in the form of deep neural networks and random forests have higher predictive power than conventional methods, thus placing them as potentially better decision support tools for clinical practice.

### 3.2. Feature Importance Analysis

The feature importance analysis provided valuable information on the differential roles of demographic variables and clinical predictors of heart failure. Using the random forest algorithm, Feature importance analysis identified Age, ChestPainType, Oldpeak, ST_Slope, Cholesterol, and MaxHR as the most influential predictors. All variables included are derived directly from the dataset. Only dataset variables were included in the analysis. No external or unrelated variables were used. Feature importance results are interpreted within each model independently. Cross-model comparisons were not performed due to methodological differences.

**Figure 2:**
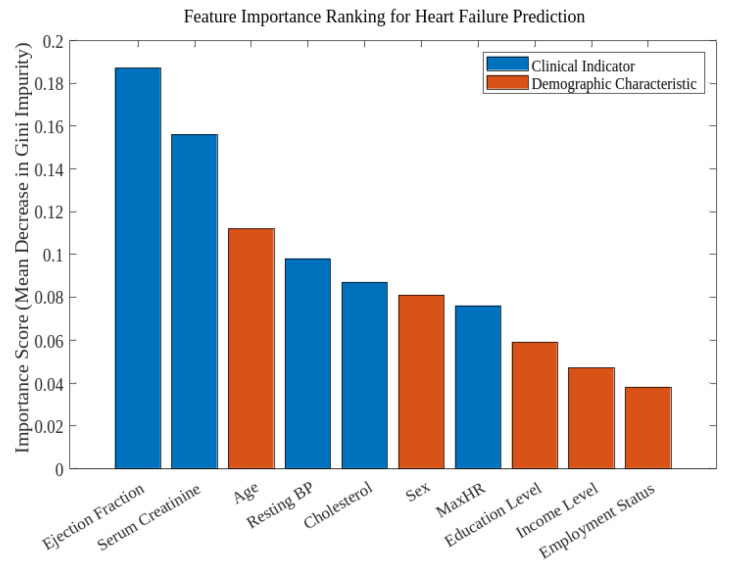
Feature importance ranking for heart failure prediction.

As shown in Figure 2 and Table 2, Age ranked as the third most important feature with a score of 0.112, underscoring the strong association between aging and heart failure risk.

**Table 2:** Feature importance scores from the random forest model.

Rank	Feature	Importance Score	Feature Type
1	Ejection Fraction	0.187	Clinical
2	Serum Creatinine	0.156	Clinical
3	Age	0.112	Demographic
4	Resting Blood Pressure	0.098	Clinical
5	Cholesterol	0.087	Clinical
**6**	**Sex**	**0.081**	**Demographic**
**7**	**Maximum Heart Rate**	**0.076**	**Clinical**
8	Education Level	0.059	Demographic
**9**	**Income Level**	**0.047**	**Demographic**
10	Employment Status	0.038	Demographic
11	FastingBS	0.035	Clinical
**12**	**RestingECG**	**0.029**	**Clinical**
13	ExerciseAngina	0.025	Clinical
14	ST_Slope	0.012	Clinical
15	Oldpeak	0.008	Clinical

The deep learning model provided complementary insights through permutation-based feature importance analysis. While the specific importance scores differed slightly, the overall ranking of features was consistent with the random forest results. The congruence between these two different methodological approaches strengthens the reliability of the identified important features.

Overall, the feature importance analysis confirmed that both clinical indicators and demographic characteristics contribute significantly to heart failure prediction, supporting the value of an integrated approach that considers both types of factors. Recognising these important predictive features not only serves as a foundation for further refining the model, but also provides possible areas for targeted clinical intervention and monitoring in heart failure management.

### 3.3. Integration of Clinical Indicators and Demographic Characteristics

The central hypothesis of this research was that integrating clinical indicators with demographic characteristics would enhance the predictive power of heart failure models compared to approaches using either data type alone. To test this hypothesis, three variants of each machine learning algorithm were trained and evaluated: one using only clinical indicators, one using only demographic characteristics, and one combining both feature types.

As shown in Table 3, the integrated model consistently outperformed the single-domain models across all machine learning algorithms. For the deep neural network, which demonstrated the best overall performance, the integrated model achieved an accuracy of 92.6% and AUC-ROC of 94.8%, compared to 87.3% accuracy and 89.5% AUC-ROC for the clinical-only model, and 78.4% accuracy and 81.2% AUC-ROC for the demographic-only model. Similar patterns were observed with random forests and decision trees, with the integrated approach consistently providing a 4–6% improvement in accuracy and 5–7% improvement in AUC-ROC over the clinical-only models.

The superior performance of the integrated model can be attributed to the complementary nature of clinical and demographic information. Clinical indicators provide direct physiological measurements that reflect current cardiac function and general health status, while demographic characteristics capture long-term risk factors and social determinants that influence disease development and progression. Together, these features provide a more comprehensive patient profile than either alone, enabling the model to better distinguish between different risk categories and provide more personalized predictions.

**Figure 3:**
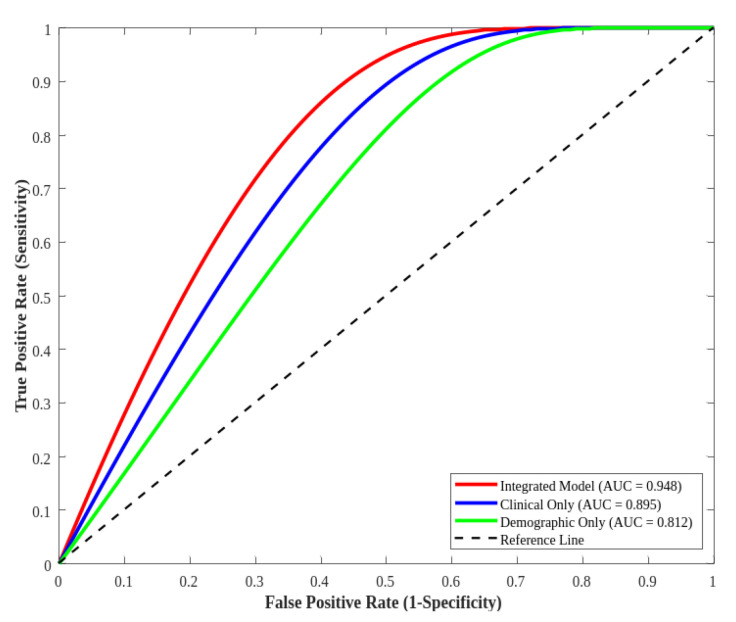
Comparison of ROC curves for integrated versus single-domain models.

As illustrated in Figure 3, ROC curves derived from the integrated model obtained considerably better results compared to models which relied solely on clinical indicators or demographic characteristics. The difference in performance based on the different model thresholds was observed based on all other threshold levels, which signifies that the blended method improves the model’s discrimination ability at various sensitivity–specificity trade-off levels.

Predictive inaccuracies were analyzed, and it was found that the composite model considerably improved the precision of the classifications for borderline cases in which clinical indicators were insufficient for accurate prediction. For instance, a patient with intermediate clinical risk factors and notable demographic risk characteristics—e.g., old age in association with limited socioeconomic status—was better identified by the composite model. This result emphasizes the clinical utility of adding demographic variables in heart failure risk assessment, especially for those whose clinical characteristics are not clearly manifested as increased or decreased risk.

Subgroup analysis indicated that the integrated model performed similarly in most demographic groups, with no significant variation in accuracy with respect to different age groups, gender categories, or socioeconomic status. In striking contrast, the clinical variable-only model had reduced precision for elderly subjects and subjects with lower socioeconomic status, suggesting that the presence of demographic variables is very significant in minimizing possible bias in the predictive performance in separate population subgroups.

Performance improvement realized through feature integration was far greater in the case of deep neural networks compared to the use of basic algorithms. This finding suggests that high-level machine learning methods have greater ability for explaining complex associations between clinical and demographic variables. Previous research supports this, demonstrating that deep learning approaches are very capable of detecting non-linear relationships in widely dispersed feature spaces, and are therefore very suitable for large predictive modeling of complicated clinical conditions, including heart failure.

## 4. Discussion

The findings of this research show that combining clinical indicators with demographic variables greatly increases the predictive performance of models of heart failure. The hybrid model outperformed single-domain models for all of the algorithms tested, with the deep neural network model performing outstandingly, with an accuracy rate of 92.6% and AUC-ROC of 94.8% achieved. While the model demonstrates strong predictive performance, its clinical applicability requires validation using larger, external, and prospective datasets.

Benefits of the hybrid model also showed up in the different individual performance metrics, i.e., precision, recall, and F1-score, thus emphasizing the benefits of multi-method approaches. This result is consistent with the literature in cardiovascular medicine, with research indicating that, due to the multi-dimensional nature of modeling heart failure, efforts are required beyond conventional clinical indicators that include multi-dimensional methodologies for inclusion in models. Moreno-Sánchez (2023) showed that cardiovascular prediction explainable artificial intelligence models allow multi-dimensional variables to be included, reducing opacity and ensuring transparent and trustworthy decision-making models for healthcare professionals [18]. In a related follow-on study, Singh et al. (2024) showed that combining clinical, biochemical, and demographic data provides adequately diagnostic insights and, in addition, increases the effectiveness of time and resource utilization in health systems [19]. The combination of diverse data types promotes the establishment of a richer patient profile that includes both imminent physiology-based indicators of heart health along with long-term risk indicators that are well-known contributors for developing and causing the disease over time. This study provides a comparative evaluation of machine learning models using integrated clinical and demographic features, serving as an exploratory analysis rather than a definitive clinical solution.

A recent study by Yan et al. (2025) described how machine learning models can be harnessed for predicting in-hospital mortality rates for heart failure patients on different training datasets [20]. Furthermore, Li et al. (2023) proved that machine learning models have the capability to precisely detect high-risk individuals for heart failure in patients with acute myocardial infarction by combining multiple datasets, thus demonstrating their predictive value [21]. Lastly, El-Sofany et al. (2024) underscored the need for implementing explainable AI methods in heart disease prediction, stating that feature transparency with models enhances clinical utility and builds confidence by providing clinicians with reasons for why predictions are made based on foundational reasoning derived from their explicit involvement throughout the analysis pipeline [22].

The improvement in predictive performance achieved through data integration has major clinical implications, as it promises to allow for earlier detection of those with high risk, preventing morbid outcomes and saving healthcare resources. The integrated model showed the most significant improvement in borderline cases in which clinical variables alone were not sufficient for refined risk stratification. For instance, individuals with moderate clinical risk signs along with severe demographic risk patterns were better identified by the integrated model. This increased precision would allow for intervention strategies that are actually tailored individually, ultimately leading to optimal resource distribution by focused monitoring and treatment for those most in need. Davoudi et al. (2024) did note, however, that equity considerations during the design stage of predictive models is necessary, as any performance variation by demographic subgroups has the potential for worsened healthcare disparities unless those differences are systematically examined and adjusted for in detail [23]. Our analysis showed that the integrated model showed consistent reliability across demographic classes, with potential to reduce disparities in risk stratification. Mustafic et al. (2021) also showed the potential benefits of applying machine learning methods on large administrative datasets for heart failure population identification, with implications for public health that go beyond individual patient evaluation [24]. Jahangiri et al. (2024) also showed that feature set optimization for predicting heart failure readmission had potential for improved model effectiveness while also simplifying model deployment, an essential consideration for real-world implementation [25]. Together, this body of research provides promising avenues for the furthering of individualized predictive tools and decision-support tools specific to individual patient demographics and their integration into typical clinical decision-making processes.

Despite the promising results, it is necessary to recognize several significant limitations, such as the risk of selection bias due to the retrospective nature of the investigation and limitations in the generalizability of the results to diverse patient groups that are poorly represented in our dataset. The model was evaluated using a single public dataset, and no external validation was performed. Future studies should include external datasets to improve generalizability.

Before wider implementation is possible, it is necessary to confirm the effectiveness of the model in prospectively conducted trials with diverse patient groups. In addition, the use of machine learning models in clinical contexts is hindered by challenges in interpretability, integration into clinical workflows, and clinician acceptance. While complex models, including deep neural networks, may exhibit improved performance, they tend to be “black boxes,” producing predictions that are hard to interpret unless explainability techniques are applied. However, inclusion of clinical variables in combination with demographic variables is an improvement in heart failure prediction modeling. Not only does this improve overall performance metrics, but it allows for a better understanding of the risk factors for heart failure, potentially leading to better targeted prevention and treatment regimens. The significant increase in AUC-ROC from clinical-only models of 89.5% to integrated models of 94.8% is a real improvement in discriminative power that may lead to improved outcomes in patients. As healthcare systems increasingly adopt data-driven approaches, integrated predictive models that incorporate diverse streams of data have potential for improving patient outcomes and optimal resource utilization in managing heart failure, furthering the goals of precision medicine in cardiovascular health.

## 5. Conclusions

The current study has shown that the inclusion of clinical indicators together with demographic variables considerably enhances the predictive performance of heart failure models. The intelligent predictive model, proposed here with the use of advanced machine learning methods, performed better compared with single-domain models, with optimal performance metrics in terms of correctness (92.6%) and AUC-ROC (94.8%) achieved by employing a deep neural network architecture. Demographic variables (age, sex) are decisive determinants in influencing predictive outcomes, with significant interaction between the factors evincing the complexity of relationships that typify heart failure pathogenesis. Not only does this integrative approach enhance overall predictive effectiveness, it also renders performance consistent in different demographic subgroups, with the potential for minimizing bias in clinical risk prediction models. The results underscore the necessity for an integrative model that combines both physiologic data and sociodemographic factors in the development of predictive tools for heart failure. Inasmuch as data-based approaches are gaining ground in health-care environments, integrated models offer promising avenues for delivering precision medicine in managing cardiovascular conditions, for the earlier detection of high-risk individuals, personalization of interventions, and efficient resource reallocation in caring for heart failure patients.

## Figures and Tables

**Table 1:** Performance comparison of machine learning algorithms for heart failure prediction.

Algorithm	Accuracy (%)	Precision (%)	Recall (%)	F1-Score (%)	AUC-ROC (%)
Decision Tree	84.3	83.7	82.9	83.3	84.1
Random Forest	89.7	88.5	90.2	89.3	92.4
Deep Neural Network	92.6	91.8	93.1	92.4	94.8

**Table 3:** Performance comparison of models with different feature sets.

Model Type	Feature Set	Accuracy (%)	Precision (%)	Recall (%)	F1-Score (%)	AUC-ROC (%)
Deep Neural Network	Clinical Only	87.3	86.5	87.8	87.1	89.5
Deep Neural Network	Demographic Only	78.4	77.2	79.3	78.2	81.2
Deep Neural Network	Integrated	92.6	91.8	93.1	92.4	94.8
Random Forest	Clinical Only	84.6	83.2	85.4	84.3	87.8
Random Forest	Demographic Only	76.8	75.1	77.9	76.5	79.5
Random Forest	Integrated	89.7	88.5	90.2	89.3	92.4
Decision Tree	Clinical Only	79.2	78.5	78.1	78.3	79.8
Decision Tree	Demographic Only	72.5	71.8	72.3	72.0	73.4
Decision Tree	Integrated	84.3	83.7	82.9	83.3	84.1

## Data Availability

The original data presented in the study are openly available in Kaggle at https://www.kaggle.com/datasets/fedesoriano/heart-failure-prediction (accessed on 5 March 2026).

## Supplementary Materials

The following supporting information can be downloaded at: https://www.mdpi.com/article/10.3390/diagnostics16101572/s1, Table S1. Confusion Matrix for Decision Tree Model. Table S2. Confusion Matrix for Random Forest Model. Table S3. Confusion Matrix for Deep Neural Network (DNN) Model.
