# Supplementary material for: Enhanced Prediction of Cardiovascular Disease Through Integrated Machine Learning Models Combining Clinical and Demographic Characteristics
Source: Diagnostics (Basel). 2026 May 21;16(10):1572. doi: 10.3390/diagnostics16101572 (PMC13205830; doi:10.3390/diagnostics16101572)
Supplement: Supplementary file 1 [file diagnostics-16-01572-s001.zip › diagnostics-4213536-supplementary.pdf]

Supplementary Table S1. Confusion Matrix for **Decision Tree Model**

Model	Predicted Positive (Heart Disease)	Predicted Negative (No Heart Disease)	Total
**True Positive (TP)**	130	18	148
**True Negative (TN)**	110	31	141
**False Positive (FP)**	15	14	29
**False Negative (FN)**	27	23	50
**Accuracy**	84.3%		
**Precision**	83.7%		
**Recall**	82.9%		
**F1-Score**	83.3%		
**AUC-ROC**	84.1%		

Supplementary Table S2. Confusion Matrix for **Random Forest Model**

Model	Predicted Positive (Heart Disease)	Predicted Negative (No Heart Disease)	Total
**True Positive (TP)**	140	16	159
**True Negative (TN)**	122	17	139
**False Positive (FP)**	14	12	26
**False Negative (FN)**	16	23	39
**Accuracy**	89.7%		
**Precision**	88.5%		
**Recall**	90.2%		
**F1-Score**	89.3%		
**AUC-ROC**	92.4%		

Supplementary Table S3. Confusion Matrix for **Deep Neural Network (DNN) Model**

Model	Predicted Positive (Heart Disease)	Predicted Negative (No Heart Disease)	Total
**True Positive (TP)**	148	11	159
**True Negative (TN)**	127	12	139
**False Positive (FP)**	12	16	28
**False Negative (FN)**	11	23	34
**Accuracy**	92.6%		
**Precision**	91.8%		
**Recall**	93.1%		
**F1-Score**	92.4%		
**AUC-ROC**	94.8%		
